# Intravitreal conbercept for chronic central serous chorioretinopathy with occult CNV: a retrospective clinical study based on multimodal ophthalmic imaging

**DOI:** 10.3389/fmed.2025.1550543

**Published:** 2025-03-25

**Authors:** Suyu Wang, Jiajun Li, Zhipeng Yan, Qin Jiang, Keran Li

**Affiliations:** Department of Ophthalmology, The Affiliated Eye Hospital of Nanjing Medical University, Nanjing, China

**Keywords:** choroid, neovascularisation, retina, vascular endothelial growth factor, treatment medical

## Abstract

**Purpose:**

This study aimed to evaluate the therapeutic efficacy and safety of intravitreal conbercept in patients with chronic central serous chorioretinopathy (cCSC) complicated by occult choroidal neovascularization (CNV), and to explore its potential in improving visual function and various ophthalmic parameters.

**Methods:**

This retrospective, longitudinal, comparative study included 50 patients diagnosed with cCSC and occult CNV. Patients underwent intravitreal conbercept injections and were monitored over a six-month period. Comprehensive ophthalmic evaluation included best-corrected visual acuity (BCVA), central macular thickness (CMT), subretinal fluid (SRF) status, subfoveal choroidal thickness (SFCT), and optical coherence tomography angiography (OCTA). OCTA parameters such as foveal avascular zone (FAZ) area and CNV lesion characteristics were analyzed pre- and post-treatment. Patients were categorized based on changes in CNV lesion size to identify prognostic factors influencing treatment response.

**Results:**

Significant improvements were observed in mean BCVA from baseline (0.78 ± 0.50 vs. 0.32 ± 0.31, *p* < 0.01) in all 50 eyes of the patients, except for one eye. Additionally, there were significant improvements in CMT, SRF status, SFCT, FAZ area, and CNV lesion size post-treatment (*p* < 0.05). Pearson correlation analysis indicated a positive correlation between baseline BCVA and CMT (*r* = 0.3615, *p* = 0.0116). Changes in BCVA post-treatment correlated with alterations in CMT, SRF diameter, and CNV lesion size. Patients with a favorable treatment response had significantly lower baseline CMT (312.17 ± 57.39 vs. 428.86 ± 114.54, *p* < 0.05) and CNV vessel diameter (17.46 ± 2.72 vs. 24.84 ± 4.02, *p* < 0.01) compared to those with unfavorable responses.

**Conclusion:**

Intravitreal conbercept injection was found to be safe and effective in improving BCVA and various ophthalmic parameters in patients with cCSC complicated by occult CNV, with no significant adverse effects observed during the study period. Baseline CMT, SRF diameter, CNV lesion size, and mean CNV vessel diameter were identified as valuable indicators for assessing treatment response and prognosis. These findings provide important insights for the clinical management and prognostic evaluation of cCSC patients with occult CNV, highlighting the utility of multimodal imaging in assessing treatment outcomes.

## 1 Introduction

Central serous chorioretinopathy (CSC) is a retinal disorder characterized by localized detachment of the neuroepithelium in the macular region or posterior pole, often accompanied by focal protrusion of the retinal pigment epithelium (RPE) (1). Pathologically, CSC is classified into acute and chronic CSC according to the disease duration. Acute CSC typically resolves spontaneously within 3–4 months with a favorable prognosis (2). However, a subset of patients experience a prolonged disease course exceeding 6 months, leading to persistent subretinal fluid (SRF) and patchy RPE atrophy, termed chronic CSC (cCSC) (3). Approximately 2–15.6% of individuals with cCSC may develop choroidal neovascularization (CNV) as a secondary complication (4).

Patients with cCSC and CNV often face poorer prognoses (5). Notably, some may also develop occult CNV, further complicating their clinical course. As a subtype of CNV, occult CNV is easily missed in routine single ophthalmic imaging modalities, such as spectral-domain optical coherence tomography (OCT), potentially delaying treatment and causing irreversible vision loss (6). Unlike classic CNV, occult CNV is less well-defined and does not show clear boundaries on FFA. It is characterized by indeterminate or poorly delineated fluorescence. It usually lies beneath the RPE and may present as fibrovascular pigment epithelial detachment (PED) or late leakage of undetermined source. Currently, the utilization of multimodal imaging is increasingly pivotal in enhancing the accuracy of disease detection (7). Within ophthalmology, multimodal imaging technologies encompass OCT combined angiography, scanning laser ophthalmoscopy (SLO), adaptive optics, photoacoustic technologies, and more (8). These advanced methods in image acquisition and processing significantly enhance resolution and imaging specificity, thereby proving invaluable for diagnosing conditions such as cCSC with occult CNV and various other ocular diseases (9, 10).

Various treatment modalities for CSC include traditional focal laser therapy, half-dose photodynamic therapy (PDT), subthreshold micropulse laser, and targeted navigated laser therapy (11–14). However, this treatment approach primarily targets patients with acute CSC or cCSC without CNV, focusing on facilitating the absorption of subretinal fluid. Studies have demonstrated that anti-vascular endothelial growth factors (VEGF) therapies, such as intravitreal bevacizumab injection, exhibit therapeutic efficacy in treating cCSC with CNV (15, 16). However, a definitive therapeutic approach for cCSC with occult CNV remains elusive. Conbercept (KH902, Chengdu Kanghong Biotech Co), a recombinant fusion protein developed in China, binds to the second, third, and fourth immunoglobulin domains of VEGF receptors 1 and 2, exhibiting high affinity for VEGF-A and VEGF-B (17). This makes conbercept a critical agent in managing ocular diseases characterized by retinal neovascularization and exudation. Extensive research supports its efficacy in neovascular age-related macular degeneration (AMD) (18–21). Given the shared pathogenesis between AMD and cCSC, conbercept holds promise for treating cCSC with occult CNV, though its use in this context is underexplored. In this study, we explored the efficacy of conbercept in treating cCSC with occult CNV.

## 2 Methods

### 2.1 Study design and patients

This retrospective study collected data on patients with cCSC and occult CNV who were treated with intravitreal conbercept injections at the Eye Hospital of Nanjing Medical University from April 2020 to December 2023. This study received ethical permission from the Ethics Committee of Nanjing Medical University Eye Hospital and adhered to the ethical principles enshrined in the Declaration of Helsinki. All the subjects were informed about the experimental methods and potential risks, and all provided written consent in the form of informed consent before the commencement of this study.

The inclusion criteria were (1) patients aged ≥18 years, (2) definitively diagnosed with cCSC with occult CNV, (3) the presence of symptoms associated with cCSC combined with occult CNV, including persistent or recurrent visual blurriness, vision loss, and metamorphopsia, (4) persistent SRF (>6 months) in the macular region or SRF recurrence of more than three times with intervals of <2 months with characteristic “double-layer sign” on OCT and accompanied by an increase in SFCT, (5) formation of CNV in the choroidal capillary layer, situated beneath the RPE layer accompanied by blood flow signals on OCT angiography (OCTA), (6) no CNV fluorescence but with punctate hyperfluorescent foci and localized leakage in the macular pigment epithelium during the venous phase of fluorescein fundus angiography (FFA), (7) dilated choroidal vessels and hyperperfusion in the affected area on Indocyanine Green Angiography (ICGA), and (8) followed-up for >6 months (22, 23).

The exclusion criteria were (1) patients with a history of severe systemic, generalized, and immune-mediated diseases, (2) with choroidal or retinal diseases such as polypoidal choroidal vasculopathy, diabetic retinopathy, central retinal vein occlusion, and AMD, (3) with suboptimal or unclear imaging quality, (4) with a history of intraocular surgery, trauma, laser photocoagulation, and other intraocular injections, and (5) recent (within 3 months) use of local or systemic glucocorticoids.

### 2.2 Treatments

Upon diagnosis of cCSC with occult CNV, all cases were initiated on intravitreal conbercept treatment (0.5 mg, Chengdu Kanghong Biotech Co.). All procedures were performed under operating room conditions. After administration of local anesthesia and sterilization, a speculum was used to maintain the eyelid in an open state, and conbercept was injected through a puncture site located 4 mm from the limbus in the superior temporal quadrant. Post-injection, topical antibiotic treatment was administered. All patients received three consecutive intravitreal conbercept injections that were administered at monthly intervals.

### 2.3 Ophthalmic examination

All patients underwent comprehensive ophthalmic evaluations at baseline (0 months) that included slit-lamp examination, intraocular pressure measurement, BCVA, OCT, OCTA, FFA, and ICGA. Subsequent data collection was conducted at 1, 2, 3, and 6 months post-initial treatment.

BCVA assessment was conducted using the Snellen chart and converted to the logarithm of minimum angle resolution (logMAR) for quantitative analysis. Eye pressure was measured using a non-contact tonometer. Ultra-widefield image was obtained with the Optos^®^ Optomap^®^ P200Tx. Using the Heidelberg Spectralis (HRA + OCT) confocal laser simultaneous angiography system, enhanced depth imaging-OCT scans were conducted to examine the retinal choroid in the macular region of the affected eye. The CMT, SFCT, and height and diameter of the SRF were accurately measured. Using the AngioVue OCTA system (Avanti RTVue XR, Optovue), the macular region of the retina was examined by scanning a 3 mm × 3 mm area, achieving an image resolution of 304 pixels × 304 pixels. Utilizing automated system processing, the retina was segmented to measure the area of the FAZ. In the neovascularization area measurement mode of AngioVue system, after marking the CNV lesion area to be measured, the algorithm automatically extracted the blood flow signal within the range, and the CNV blood flow area value was automatically counted and expressed in millimeters (mm^2^). ImageJ software^1^ was used to quantify the mean CNV vessel diameter on OCTA-derived images. Images from FFA and ICGA examinations were captured during the early (2–3 min post-injection), intermediate (5–10 min post-injection), and late (10–15 min post-injection) angiographic phases. Leakage points and abnormal neovascular patterns were identified in the early phase, while the evolution of leakage was carefully monitored in the late phase.

### 2.4 Statistical analysis

The mean and standard deviation (SD) of each patient’s examination data were calculated, with continuous variables expressed as mean ± SD. Statistical analysis and figure generation were conducted utilizing GraphPad Prism 9.0 software. Using one-way repeated measures analysis of variance, we analyzed differences in ocular examination data across all follow-ups. Paired t-tests compared data between specific time points, and Pearson’s correlation assessed linear relationships between variables. The missing values were imputed using the last observation carried forward (LOCF) method. *p* values of <0.05 were considered statistically significant.

## 3 Results

### 3.1 General clinical characteristics and baseline data

This study included 50 eyes from 50 patients (29 men with 29 eyes and 21 women with 21 eyes). The patients’ ages ranged from 31 to 65 years, with a mean of 51.49 ± 7.32 years. The baseline characteristics of the participants are summarized in Table 1. During the follow-up period, only one eye did not show improvement in clinical ophthalmic indicators. No ocular adverse events, such as endophthalmitis, intraocular inflammation, and elevated IOP, or non-ocular adverse events, such as thromboembolic events and hypertension, were reported. Close attention remains to be paid during prolonged follow-up.

**Table 1:** Baseline characteristics of patients.

Parameters	Characteristics
	*N* = 50 (eyes, *N* = 50)
Mean age (years)	51.49 ± 7.32
Sex (male/female)	29/21
BCVA (logMAR)	0.78 ± 0.50
IOP (mmHg)	15.81 ± 2.73
Central macular thickness (μm)	364 ± 90.34
Subretinal fluid height (μm)	139 ± 76.95
Subretinal fluid diameter (μm)	1,528 ± 777.2
Foveal avascular zone area (mm^2^)	0.41 ± 0.073
Choroidal neovascularization area (mm^2^)	0.44 ± 0.35
Sub-foveal choroidal thickness (μm)	376.7 ± 44.05

### 3.2 Changes in ophthalmic examination data before and after treatment

**Figure 1:**
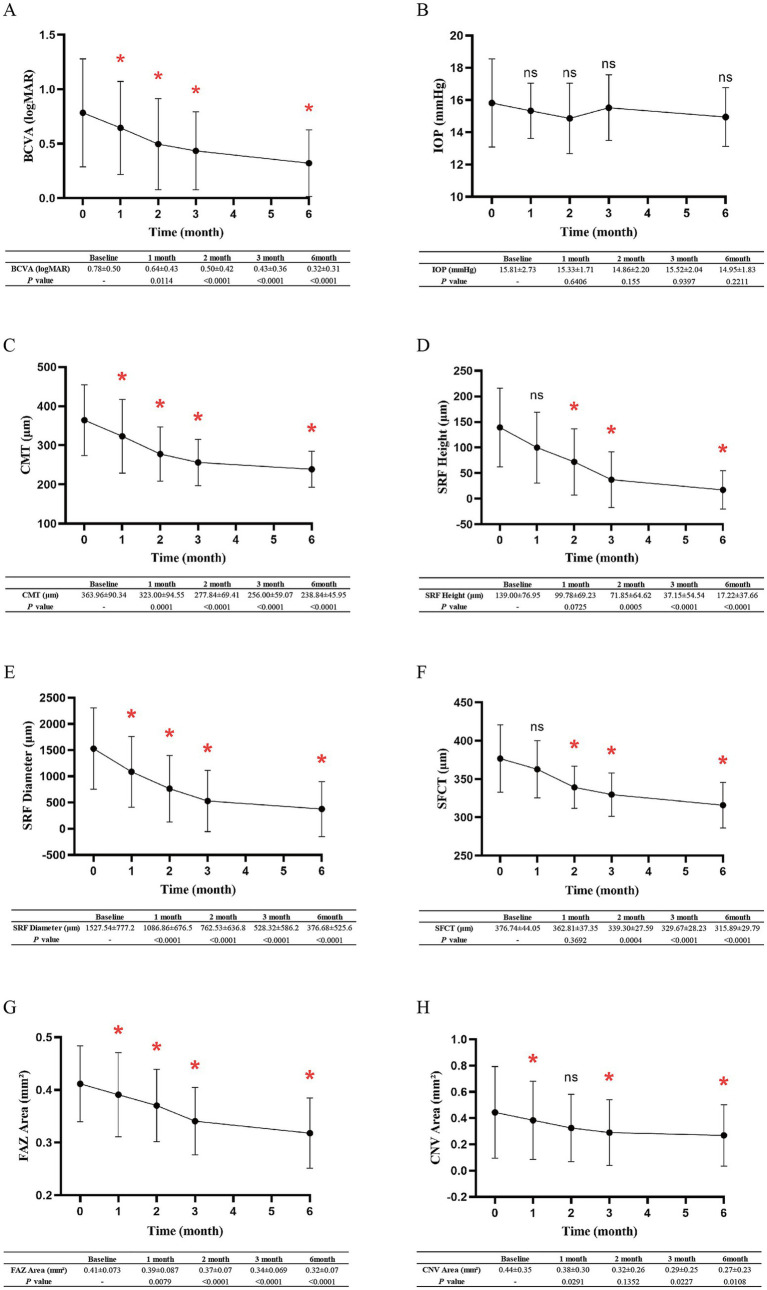
Mean changes in ophthalmic examination data across various follow-ups. **(A–H)** Mean changes in BCVA, IOP, CMT, SRF height, SRF diameter, SFCT, FAZ area, and CNV area of patients from baseline to 6 months. Error bars denote standard deviation. * indicates *p*-value < 0.05, denoting a statistically significant difference.

During the follow-up period, the average BCVA gradually improved. Compared to baseline, the average BCVA was significantly higher at the final follow-up (0.78 ± 0.50 vs. 0.32 ± 0.31, *p* < 0.01), while intraocular pressure (IOP) did not significantly change (15.81 ± 2.732 mmHg vs. 14.95 ± 1.830 mmHg, *p* = 0.2211) (Figures 1A,B). OCTA examination revealed that, following treatment, patients exhibited a significant reduction in CMT compared to that at baseline (364 ± 90.34 μm vs. 238.8 ± 45.95 μm, *p* < 0.01) (Figure 1C). The decrease is directly attributed to the absorption of SRF; 71% (20/28) of the affected eyes exhibited complete absorption of SRF within 6 months post-treatment. At the final follow-up, OCT demonstrated a marked decrease in both the average height and diameter of SRF compared to baseline (Figures 1D,E). Additionally, after 6 months of treatment, SFCT values significantly decreased compared to those at baseline (376.7 ± 44.05 μm vs. 315.9 ± 29.79 μm, *p* < 0.01) (Figure 1F).

On 3 × 3-mm OCTA images, the FAZ area was enlarged in patients with cCSC accompanied by occult CNV. After conbercept treatment, the mean FAZ area decreased from that at baseline (0.41 ± 0.073 mm^2^ vs. 0.32 ± 0.07 mm^2^, *p* < 0.01) (Figure 1G). Notably, in the initial stages of treatment, the mean CNV area exhibited a trend of reduction among patients, albeit with limited consistency (0.44 ± 0.35 mm^2^ vs. 0.32 ± 0.26 mm^2^, *p* = 0.1352). After 3 months, a significant reduction in the CNV area was observed (0.44 ± 0.35 mm^2^ vs. 0.27 ± 0.23 mm^2^, *p* = 0.0108) (Figure 1H).

**Figure 2:**
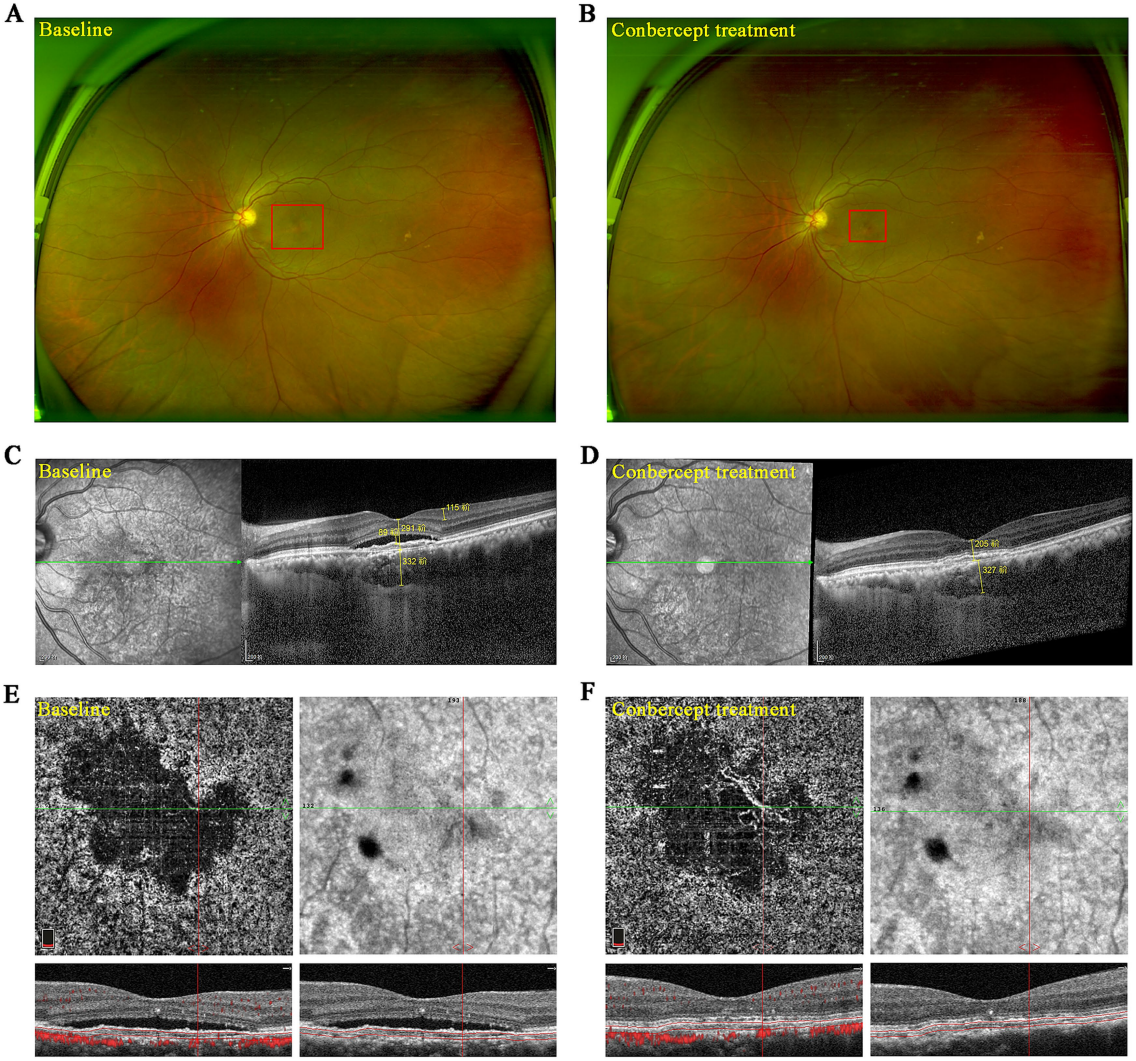
Multimodal ophthalmic imaging of one patient with cCSC and occult CNV before and after conbercept treatment. **(A,B)** Pre- and post-treatment ultra-widefield fundus images. **(C,D)** Pre- and post-treatment images of OCT. **(E,F)** Pre- and post-treatment images of OCTA.

Figure 2 presents the multimodal ophthalmic images of a 53-year-old female patient before and after treatment. After three injections of conbercept, ultra-widefield imaging revealed a reduction in lesion area (Figures 2A,B), while OCT indicated a decrease in retinal thickness (Figures 2C,D). OCTA confirmed the restoration of retinal structure while no significant improvement was noted in occult CNV (Figures 2E,F).

### 3.3 Sustained therapeutic effectiveness

**Figure 3:**
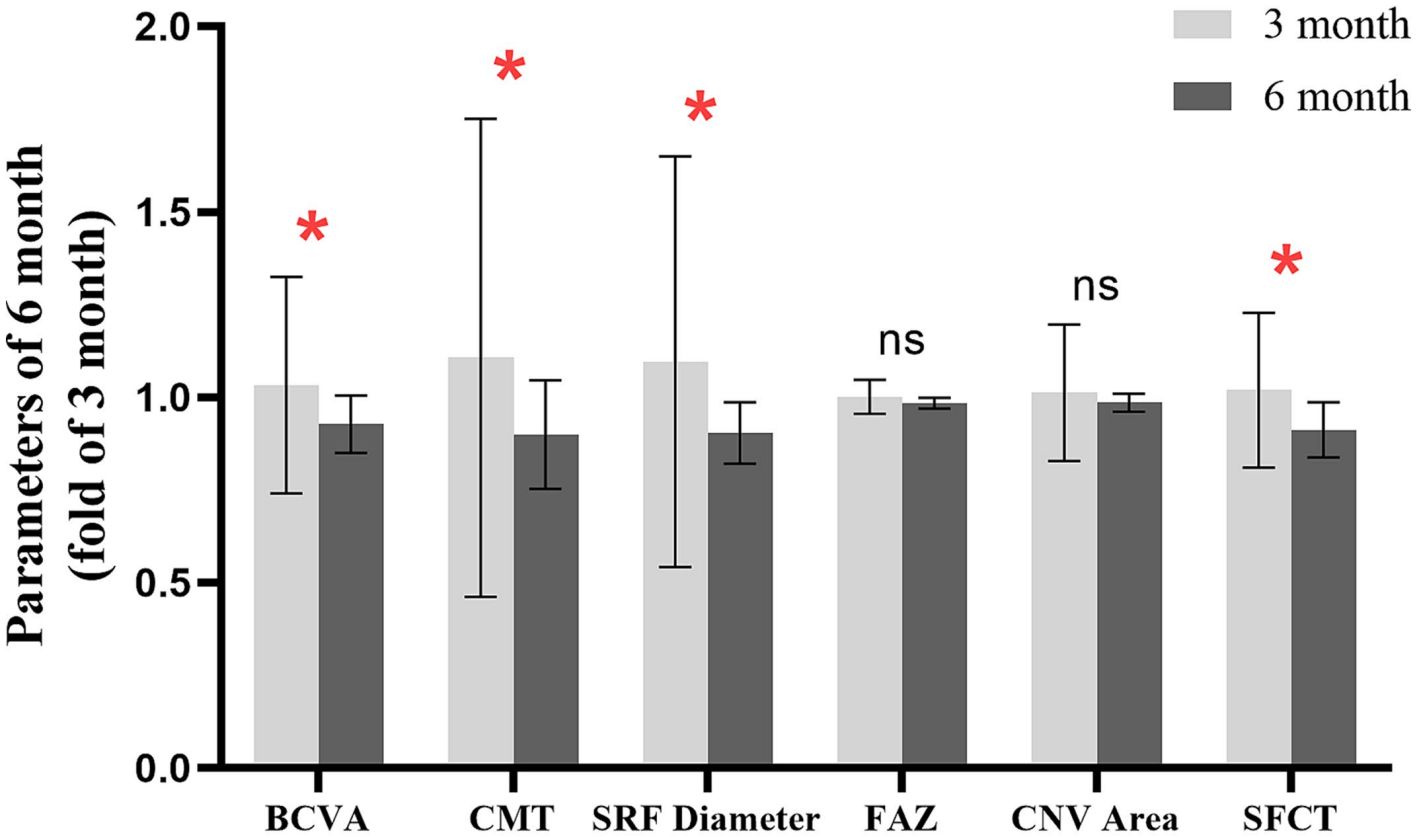
Changes in clinical indices in the post-treatment phase. * indicates *p*-value < 0.05, denoting a statistically significant difference.

Based on the medication cycle and frequency, we divided the follow-up period into a treatment phase (1–3 months) and a post-treatment phase (3–6 months). By comparing various clinical indicators at 3 and 6 months, we find that there was a sustained improvement in BCVA, CMT, SRF diameter, and SFCT following conbercept treatment. Although changes in FAZ and CNV areas did not reach statistical significance, they demonstrated a trend toward improvement, with no observed rebound phenomenon (Figure 3).

### 3.4 Correlation between visual prognosis and other clinical indices

**Figure 4:**
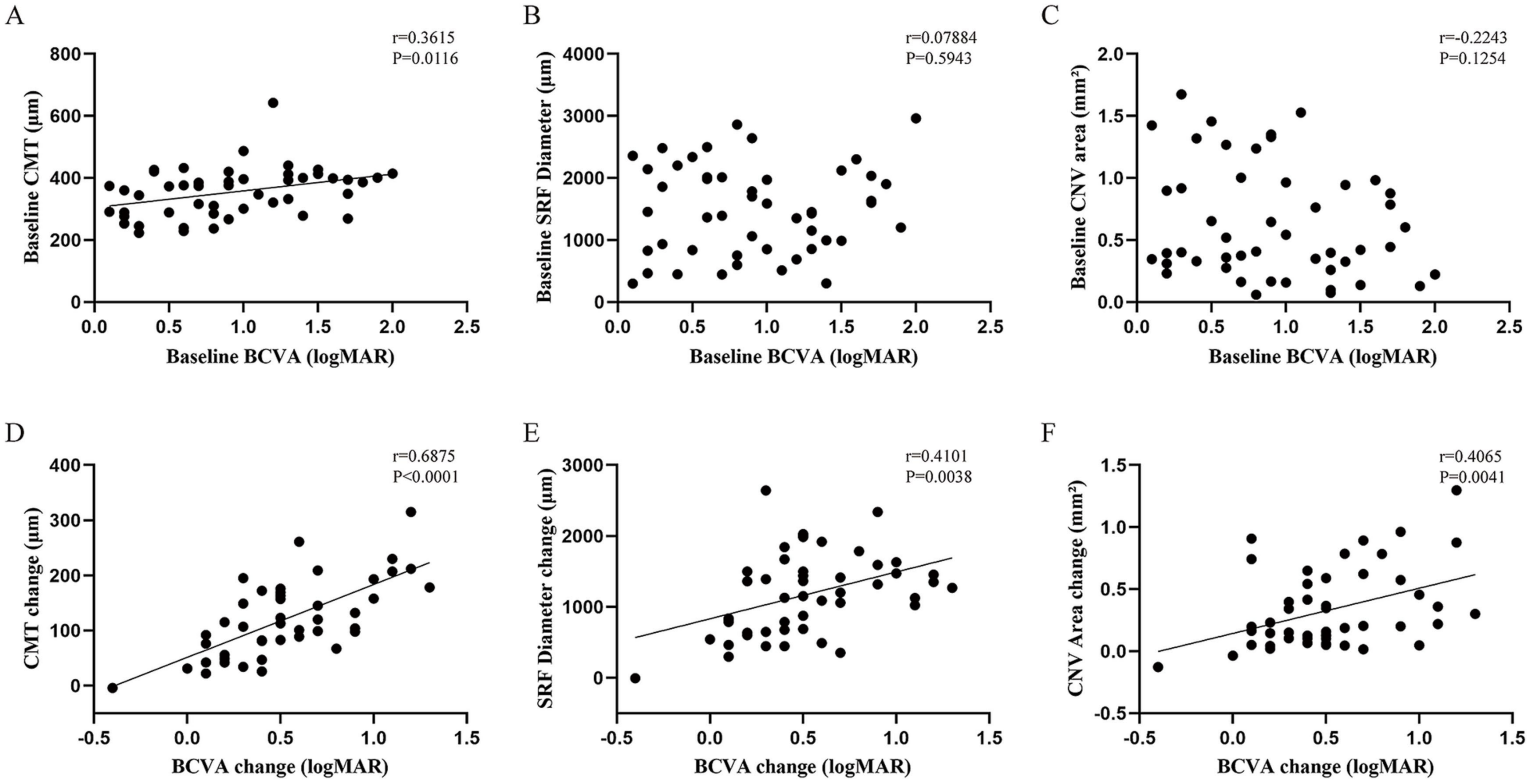
Correlation analysis of BCVA with clinical indicators. **(A)** At baseline, BCVA positively correlated with CMT. **(B,C)** At baseline, BCVA did not correlate with the CNV area or SRF diameter. **(D–F)** Change in BCVA before and after treatment positively correlated with changes in CMT, SRF diameter, and CNV area.

Using Pearson correlation analysis, this study examined the relationship between BCVA and other ocular parameters in patients with cCSC complicated by occult CNV. Initially, a positive correlation was observed between BCVA and central macular thickness (CMT) at baseline (*r* = 0.3615, *p* = 0.0116), whereas no significant correlations were found with other indices. This indicates a direct association between BCVA and CMT upon initial assessment (Figures 4A–C). Furthermore, we analyzed the changes in BCVA relative to changes in other ocular parameters before and after treatment. Analysis revealed a positive correlation between improvements in BCVA and reductions in CMT (*r* = 0.6875, *p* < 0.0001), as well as changes in SRF diameter (*r* = 0.4101, *p* = 0.0038) and CNV lesion area (*r* = 0.4065, *p* = 0.0041). These findings suggest that enhancements in CMT, SRF diameter, and CNV area are closely linked to visual prognosis in patients, potentially serving as predictive indicators of treatment outcomes (Figures 4D–F).

### 3.5 Response of the CNV area to conbercept treatment

Based on the degree of improvement in the choroidal neovascularization (CNV) area, patients achieving a reduction of >60% were classified as “well-responders,” while those with a reduction of <40% were classified as “poor-responders.” This study included 21 well-responders and 17 poor-responders. Baseline comparisons revealed no significant differences in age, BCVA, or FAZ between the groups, but the poor-responder group exhibited higher CMT (312.17 ± 57.39 vs. 428.86 ± 114.54, *p* < 0.05) and a larger mean CNV vascular diameter (17.46 ± 2.72 vs. 24.84 ± 4.02, *p* < 0.01) (Table 2).

**Table 2:** Comparison of clinical indicators across response groups.

Parameters (Baseline)	Well-responders (*N* = 21)	Poor-responders (*N* = 17)	*P* value
Sex (male/female)	13/8	9/8	-
Mean age (years)	51.17 ± 7.81	54.57 ± 7.46	0.3649
BCVA (logMAR)	0.81 ± 0.54	0.73 ± 0.49	0.7517
Central macular thickness (μm)	312.17 ± 57.39	428.86 ± 114.54	0.0083*
Foveal avascular zone area (mm^2^)	0.39 ± 0.06	0.45 ± 0.10	0.1471
CNV vessel diameter (μm)	17.46 ± 2.72	24.84 ± 4.02	0.0002*

## 4 Discussion

CSC is a choroidal thickening spectrum disorder characterized by choroidal thickening and choroidal vascular hyperplasia (24). As cCSC progresses, its combination with CNV may result in irreversible visual loss, while few studies have proposed definitive treatment strategies (25). Several studies have shown that ranibizumab and aflibercept are effective in reducing subretinal fluid (SRF) and improving visual acuity in patients with cCSC and CNV. However, the response to ranibizumab can be variable, and some patients may require multiple injections to achieve sustained benefits, and because of the potential for recurrence, long-term efficacy remains uncertain (26, 27). Half-dose PDT is considered a first-line treatment for cCSC. Studies have shown that PDT can effectively reduce SRF and improve visual acuity by targeting the choroidal hyperpermeability that underlies cCSC. However, it has no significant effect on CNV area in cCSC with CNV (28). Combination PDT and anti-VEGF is effective for chronic CSC which has failed conventional therapy (29). Conbercept effectively suppresses the interaction between VEGF and its receptors, exhibiting multiple targeting, strong affinity, and prolonged intraocular activity (30). Conbercept has shown promise in various retinal diseases, yet its role in treating cCSC with occult CNV remains underexplored. In this study, nearly all (49 out of 50) patients diagnosed with cCSC complicated by occult CNV demonstrated significant improvement in BCVA following three intravitreal injections of conbercept. Additionally, through a comprehensive analysis of diverse ophthalmic examination parameters utilizing integrated multimodal ophthalmic imaging, significant improvements were noted in CMT, SRF, SFCT, FAZ, and CNV. These findings underscore the efficacy of the integrated imaging approach in evaluating and monitoring ocular health, highlighting conbercept’s ability to mitigate fluid accumulation and vascular abnormalities associated with cCSC and occult CNV. Importantly, improvements in ocular parameters persisted beyond the treatment period, indicating the safety, efficacy, and stability of conbercept in treating cCSC with occult CNV.

On the other hand, analysis revealed a close relationship between the CMT level and BCVA, suggesting that the degree of CMT improvement significantly impacts visual prognosis. The macula resides centrally within the retina and harbors a densely packed cluster of cone photoreceptors. This anatomical specialization is critical for high-acuity vision tasks, such as reading and facial recognition, owing to its role in daylight and fine-detail visual perception (31). In patients with cCSC accompanied by occult CNV, CMT elevated compared to that in normal individuals and is associated with SRF (32). As treatment progresses, gradual absorption of fluid leads to structural and functional restoration of the macular fovea. Therefore, CMT may be a key indicator that reflects the therapeutic efficacy. Following 6 months of conbercept treatment, 29% of patients still exhibited residual fluid, implying that the formation of SRF may stem not only from CNV leakage but also from choroidal vascular hyperpermeability and diffuse RPE decompensation (33, 34).

The pathogenesis of CNV associated with cCSC remains unknown. Previous perspectives postulated that prolonged retention of SRF and pigment epithelial detachment may cause decompensation of RPE and disruption of the RPE/Bruch’s membrane complex, subsequently triggering the occurrence of CNV (35, 36). Another theory suggests that in patients with cCSC, inadequate perfusion of choroidal capillaries results in ischemia and hypoxia, which stimulates the release of VEGF, thereby triggering the development of CNV. In turn, this can further lead to focal RPE dysfunction and compromise the tight junctions of endothelial cells, resulting in exudation, choroidal vessel dilation, and thickening, ultimately culminating in CNV (37, 38).

The response of the CNV area to treatment in patients with cCSC accompanied by occult CNV was less sensitive compared to that of other ocular indices. This may be attributed to the prolonged duration and higher vascular maturity of occult CNV. In this study, we found that the poor-responsers exhibited significantly larger CNV vascular diameters compared to the good-responsers. Spaide et al. proposed two distinct mechanisms of neovascularization, namely arteriogenesis and angiogenesis. Unlike VEGF-dependent angiogenesis in AMD, arteriogenesis features vascular dilation and a weak VEGF correlation and is linked to the regulation of extravascular matrix remodeling in response to endothelial shear stress (39). In thick choroidal diseases such as cCSC, choroidal hyperemia leads to increasing choriocapillaris shear stress, extracellular remodeling, and consequent damage to RPE and Bruch’s membrane (40, 41). This may explain the varying responses to conbercept treatment among patients with cCSC accompanied by occult CNV, indicating that angiogenesis and arteriogenesis play distinct roles in the progression of CNV.

Research has introduced the concept of quiescent CNV in thick choroidal neovascular diseases, which characterized by the absence of exudation and minimal impact on vision (42). As the duration of CNV cannot be uniformly determined in patients initially diagnosed with CSC accompanied by occult CNV, some may exhibit quiescent CNV or CNV entering a quiescent state (43). The development of this condition is associated with the disruption of the RPE barrier function, which makes anti-VEGF treatments less effective; this could account for the patients’ delayed rate of healing in the CNV area. Therefore, early diagnosis and accurate CNV status assessment allow for tailored treatment plans that address the specific needs of patients with CSC. Advanced imaging technology based on multimodal images and a variety of image analysis systems combined with deep learning have improved the ability to detect subtle changes in retinal and choroidal structures, which is helpful for early intervention of diseases (44, 45). However, another study in AMD with quiescent CNV suggest a potential for CNV reactivation (46). Thus, the selection of anti-VEGF drugs or combination with photodynamic therapy (PDT) remains necessary. A study on AMD with CNV revealed a close correlation between the presence of CNV’s peripheral loop and the maintenance of anti-VEGF therapy (47). Following anti-VEGF treatment, the initial CNV pattern typically transitions to a more mature form, which poses a lesser threat to vision. Therefore, anti-VEGF therapy achieves the goal of delaying disease progression, to a certain extent, by stabilizing CNV state.

While our study provides evidence of conbercept’s efficacy, several limitations warrant consideration. Firstly, the relatively small sample size (50 patients) may limit the generalizability of our findings. Larger, multicenter studies are needed to validate the efficacy and safety of conbercept in a broader patient population. Then, the single-center and single treatment regimen design of our study may introduce bias and limit the external validity of our results. Future studies should include multiple centers and comparisons of established therapies to enhance the robustness of the findings. Additionally, the six-month follow-up period is relatively short for assessing the long-term efficacy and safety of conbercept. Longer follow-up studies are necessary to determine the durability of the treatment effects and to monitor for potential late-onset adverse events.

In conclusion, our study demonstrates that intravitreal conbercept is a safe and effective treatment for cCSC complicated by occult CNV. We observed significant improvements in BCVA, CMT, SRF status, and CNV lesion size over a six-month follow-up period. These findings suggest that conbercept may offer a viable therapeutic option for this challenging condition.

## Data Availability

The original contributions presented in the study are included in the article/supplementary material, further inquiries can be directed to the corresponding authors.

## Footnotes

^1^ https://imagej.net
